# iNKT Cells Suppress the CD8^+^ T Cell Response to a Murine Burkitt’s-Like B Cell Lymphoma

**DOI:** 10.1371/journal.pone.0042635

**Published:** 2012-08-07

**Authors:** Ryan L. Bjordahl, Laurent Gapin, Philippa Marrack, Yosef Refaeli

**Affiliations:** 1 Integrated Department of Immunology, National Jewish Health and University of Colorado Denver, Denver, Colorado, United States of America; 2 Howard Hughes Medical Institute; Chevy Chase, Maryland, United States of America; 3 Charles C. Gates Center for Regenerative Medicine and Stem Cell Biology, University of Colorado School of Medicine, Aurora, Colorado, United States of America; 4 Department of Dermatology, University of Colorado School of Medicine, Aurora, Colorado, United States of America; Mayo Clinic, United States of America

## Abstract

The T cell response to B cell lymphomas differs from the majority of solid tumors in that the malignant cells themselves are derived from B lymphocytes, key players in immune response. B cell lymphomas are therefore well situated to manipulate their surrounding microenvironment to enhance tumor growth and minimize anti-tumor T cell responses. We analyzed the effect of T cells on the growth of a transplantable B cell lymphoma and found that iNKT cells suppressed the anti-tumor CD8^+^ T cell response. Lymphoma cells transplanted into syngeneic wild type (WT) mice or Jalpha18^−/−^ mice that specifically lack iNKT cells grew initially at the same rate, but only the mice lacking iNKT cells were able to reject the lymphoma. This effect was due to the enhanced activity of tumor-specific CD8^+^ T cells in the absence of iNKT cells, and could be partially reversed by reconstitution of iNKT cells in Jalpha 18^−/−^ mice. Treatment of tumor-bearing WT mice with alpha -galactosyl ceramide, an activating ligand for iNKT cells, reduced the number of tumor-specific CD8^+^ T cells. In contrast, lymphoma growth in CD1d1^−/−^ mice that lack both iNKT and type II NKT cells was similar to that in WT mice, suggesting that type II NKT cells are required for full activation of the anti-tumor immune response. This study reveals a tumor-promoting role for iNKT cells and suggests their capacity to inhibit the CD8^+^ T cell response to B cell lymphoma by opposing the effects of type II NKT cells.

## Introduction

Along with CD4^+^ FoxP3^+^ regulatory T cells, natural killer T cells (NKT cells) often play important regulatory roles in T cell responses to cancer and infection [1], [2]. NKT cells are a sub-population of alpha/beta-T cells that recognize lipid antigens presented by the non-classical MHC molecule, CD1d. Found in both mice and humans, NKT cells exert important immunoregulatory and effector functions and can express a variety of cytokines rapidly upon activation [3]. Although originally defined as NK1.1-expressing T cells, NKT cells contain both NK1.1^+^ and NK1.1^−^ populations [4]–[5]. NKT cells can be divided into two major populations based on their antigen reactivity and TCR expression. Semi-invariant NKT (iNKT) cells represent the predominant NKT cell subset in mice and humans and are defined by the expression of a semi-invariant T cell receptor (TCR). iNKT cell TCRs contain an invariant TCRalpha rearrangement (Valpha14-Jalpha18 in mice, Valpha24-Jalpha18 in humans) paired with a limited collection of TCRbeta chains (Vbetas 8.2, 7 and 2 in mice, Vbeta11 in humans) [3]. These TCRs react with the marine sponge-derived antigen, a-Galactosylceramide, and other glycolipids presented by CD1d [6], [7]. Type II NKT cells are also CD1d-restricted but their TCRs do not utilize the invariant TCRalpha chain present in iNKT cells and do not respond to alphaGalCer [2], [5]. Type II NKT cells are less well understood than iNKT cells due to the lack of a well defined cell surface marker or genetic system for their study.

iNKT cells are usually associated with the activation of the anti-tumor immune response, and Jalpha18^−/−^ mice, which specifically lack iNKT cells, have increased cancer susceptibility in several cancer models (reviewed in [2]). In addition, in vivo stimulation of iNKT cells with alpha-GalCer induces a potent anti-tumor immune response [8], [9] that is thought to be mediated through their production of IFNgamma, thereby promoting the antitumor functions of NK and CD8^+^ T cells [2], [10], [11]. In contrast to iNKT cells, type II NKT cells are typically associated with inhibition of the anti-tumor immune response [2], [12]–[14]. Work in several tumor models has demonstrated that the immune-activating effects of iNKT cells and the immunosuppressive effects of type II NKT cells can occur simultaneously [13], [14], suggesting that antagonism between iNKT and type II NKT is an important mechanism regulating anti-tumor immune responses, and that counter-regulation by NKT cells may be important for balancing self- or tumor-reactive immune responses.

Burkitt’s lymphoma (BL) is an aggressive B cell lymphoma. In 85% of cases it is caused by a translocation of the *MYC* transcription factor that places it under the influence of the immunoglobulin heavy or light chain enhancer (t8;14 or t8;22). The t(8;14) translocation has been modeled experimentally in the E-mu-MYC mouse [15], a valuable tool for the study of B cell lymphoma development and MYC oncogene function. Crossing the E-mu-MYC transgene into mice transgenic for the B cell receptor specific for hen egg lysozyme (BCR^HEL^) and expressing the HEL antigen yields lymphomas that are more aggressive than primary E-mu-MYC lymphomas. These E-mu-MYC/BCR^HEL^/sHEL lymphomas (TBLs – Triple Transgenic B cell Lymphoma) most closely resemble human BL [16].

We sought to investigate the T cell response to such a B cell lymphoma. Using a murine model of Burkitt’s lymphoma, we found that iNKT cells surprisingly promote lymphoma growth by limiting the anti-tumor CD8^+^ T cell response. In the absence of iNKT cells, CD8^+^ T cell responses lead to tumor clearance and enhanced tumor-free survival. Further, we demonstrate that stimulation of iNKT cells in tumor-bearing mice with alpha-GalCer leads to enhanced suppression of CD8^+^ T cells. Finally, experiments in CD1d1^−/−^ mice suggest that type II NKT cells positively affect the anti-tumor CD8^+^ T cell response. Our data suggest a role-reversal for both NKT cell subsets where iNKT cells suppress and type II NKT cells enhance anti-tumor immunity in B cell lymphoma. The results presented here may challenge the utility of iNKT activating therapies for patients with B cell lymphoma, and suggest that iNKT and type II NKT cells can exert opposing effects on the anti-tumor T cell response.

## Results

### Mice Deficient in αβ T Cells have Reduced Tumor Burdens

**Figure 1:**
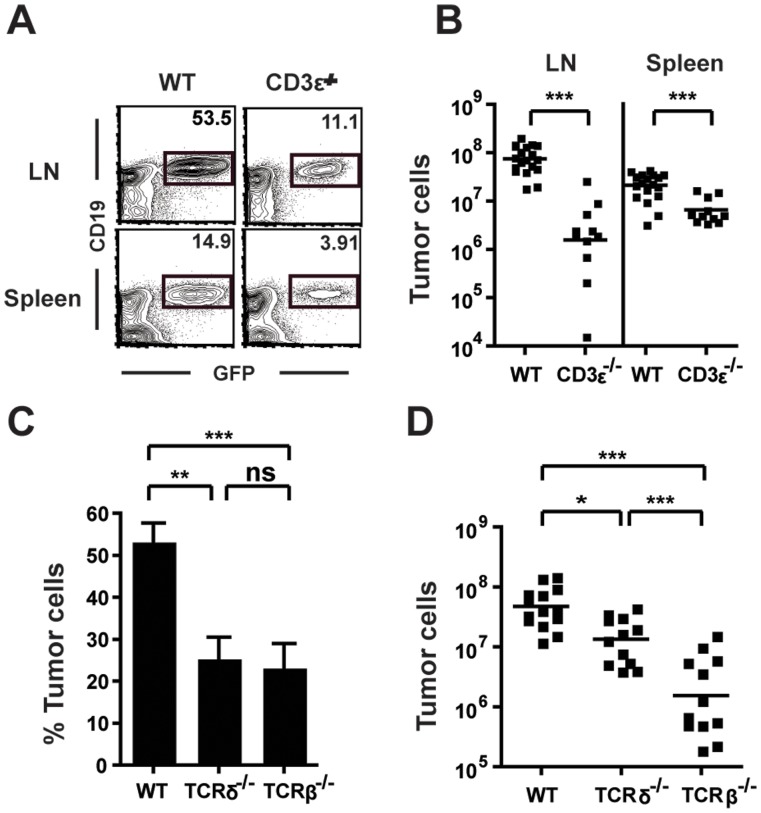
T cells promote tumor growth. (A and B) WT and CD3epsilon^−/−^ mice were analyzed 14 days after tumor transplant. Spleen and pooled LN (inguinal, axillary, brachial) cells were stained for flow cytometry. (A) Plots are gated on live cells and numbers indicate the frequency of GFP^+^CD19^+^ tumor cells present in each organ. (B) The number of tumor cells in the LN and spleen in WT and CD3epsilon^−/−^ mice was determined by flow cytometry (n = 11–19 pooled from at least 3 independent experiments). (C and D) The frequency (C) and number (D) of tumor cells present in the LN on day 14 was determined by flow cytometry for WT, TCRdelta^−/−^, and TCRbeta^−/−^ mice (n = 13–14 pooled from 3 independent experiments). *p<.05, **p<.01, ***p<.001.

To assess the role of T cells in response to systemic B cell lymphoma, we used a system in which GFP-expressing tumor cells were transplanted into cohorts of T cell deficient mice. We analyzed CD3-epsilon^−/−^ mice for tumor growth following transplantation of 10^5^ GFP^+^ TBL (Triple-Transgenic B cell Lymphoma) lymphoma cells, which are derived from an E-mu-MYC/BCR^HEL^/sHEL transgenic mouse [16], [17]. Fourteen days after transplant, tumor growth was quantified in the lymph nodes (LN, pooled inguinal, axillary, and brachial) and spleen by cell counts and flow cytometry. Surprisingly, tumor burden was reduced in both the LNs and spleens of CD3epsilon^−/−^ mice compared to WT controls (Figure 1). This was apparent in both the frequency (Figure 1A) and number (Figure 1B) of tumor cells. The absence of T cells resulted in an 18-fold decrease in tumor cells in the LN and a 3-fold decrease in the spleen. Similar results were observed upon transplantation into Rag-1^−/−^ mice (data not shown).

To define more precisely which subset of T cells was responsible for the increased lymphoma growth seen in T cell sufficient animals, we first assessed tumor growth in TCRbeta^−/−^ and TCRdelta^−/−^ mice. Tumor growth was significantly reduced in TCRbeta^−/−^ mice (Figure 1C), to an extent similar to that in CD3epsilon^−/−^ mice. Interestingly, we observed intermediate tumor growth in the TCRdelta^−/−^ strain. These mice had a reduction in tumor growth in LNs, and the numbers of tumor cells in LNs were statistically different from those in both WT and TCRbeta^−/−^ mice (Figure 1C). These data may suggest that while gamma-delta T cells support tumor growth in this model, they do so to a lesser degree than alpha-beta T cells. Because loss of alpha-beta T cells resulted in significantly fewer tumor cells compared to both WT and TCRdelta^−/−^ strains (Figure 1D), we focused further efforts on this T cell population.

### iNKT Cells are Required for Tumor Growth

**Figure 2:**
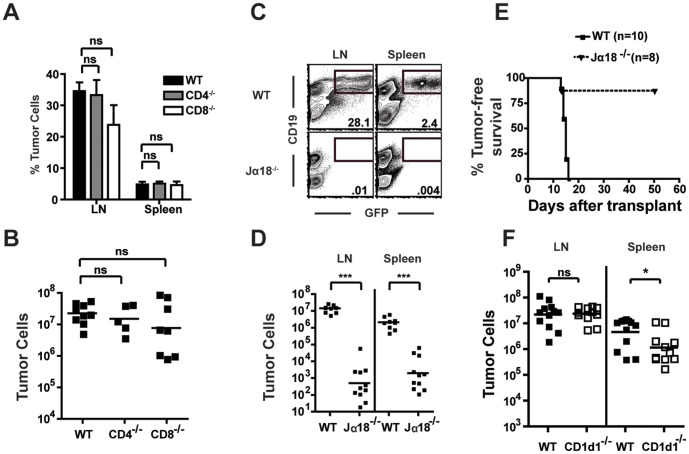
iNKT cells promote tumor growth. (A and B) The (A) frequency and (B) number of tumor cells was calculated for LN (and spleen in A) from WT, CD4^−/−^, and CD8^−/−^ mice on day 14 after lymphoma transplant (n = 5–9 pooled from 2 independent experiments). (C) LN and spleen cells from tumor-transplanted WT and Jalpha18^−/−^ mice were analyzed by flow cytometry for the presence of tumor cells. The numbers on the plots indicate the percentage of all **TCRbeta^-^**CD11b^-^ cells that were GFP^+^CD19^+^ tumor cells. (D) The numbers of tumor cells present in the LN and spleens of WT and Jα18^−/−^ mice were determined by flow cytometry (n = 9–11 pooled from three independent experiments). (E) Tumor-free survival of WT and Jalpha18^−/−^ mice was assessed (n = 8–10 from two experiments, p = .008). (F) WT and CD1d1^−/−^ mice were transplanted with 10^5^ TBL-GFP cells. Fourteen days later, the number of tumor cells present in the spleen and LN was determined. *p<.05, ***p<.001.

To characterize further the alpha-beta-T cell subtype responsible for the promotion of tumor growth, we analyzed tumor growth in CD4^−/−^ and CD8^−/−^ mice. To our surprise, the absence of either CD4^+^ or CD8^+^ T cells had no effect on tumor growth (Figure 2A–B), suggesting that neither cell type alone was critical for lymphoma growth. Because iNKT cells in mice are either double negative (DN) or CD4^+^, but not CD8^+^, and are present in both CD4^−/−^ and CD8^−/−^ mice [18], these data raised the possibility that iNKT cells might play a role in potentiating tumor growth. To test this hypothesis, we transplanted B cell lymphoma cells into iNKT cell deficient, Jalpha18^−/−^ mice and analyzed tumor development 14 days later. Tumor growth in Jalpha18^−/−^ mice was tremendously reduced compared to that in WT mice (Figure 2C–D). Further, while all WT mice (10/10) developed tumors by 15 days post-transplant, most Jα18^−/−^ mice (7/8) remained tumor free for at least 50 days (p = .008) (Fig. 2E), suggesting that the presence of iNKT cells is critical for the growth of the transplanted lymphoma.

To further examine the role of NKT cells in tumor growth we utilized a second NKT cell-deficient strain, CD1d1^−/−^ mice. These mice lack both iNKT and type II NKT cells due to the absence of CD1d, the NKT cell selecting ligand. After tumor transplant into CD1d1^−/−^ mice, tumor growth in these mice was nearly identical to WT mice, although there was a small but significant decrease in tumor growth in the spleens of CD1d1^−/−^ mice when compared with WT mice (Figure 2F). Because CD1d1^−/−^ mice lack iNKT cells like Jalpha18^−/−^, these data were surprising considering the marked decrease in tumor growth in Jalpha18^−/−^ mice and suggested that, in this system, type II NKT cells may have anti-tumor functions that counteract the pro-tumor effects of iNKT cells.

**Figure 3:**
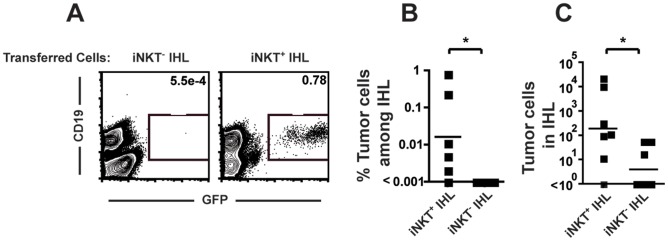
Reconstitution of iNKT cells promotes tumor growth in Jalpha18^−/−^ mice. (A) Liver mononuclear cells from WT (iNKT) and Jalpha18^−/−^ mice (Control) were adoptively transferred into Jalpha18^−/−^ recipients. 10^5^ lymphoma cells were transplanted one day later and tumor growth in the liver was analyzed on day 10 post-transplant. Representative flow plots are gated on CD11b^-^Gr1^-^CD4^-^CD8^-^ cells, and the number on the plot represents the percentage of gated cells that were CD19^+^GFP^+^ tumor cells. (B-C) The frequency (B) of tumor cells among all IHL and the absolute number of tumor cells (C) from a total of 6–7 mice in three independent experiments are shown. *p<.05.

To confirm that iNKT cells were promoting in the growth of B-cell lymphoma in vivo we transferred total leukocytes from the livers of wild type or Jalpha18^−/−^ mice into Jα18^−/−^ recipient mice. Liver mononuclear cells from wild type mice are highly enriched for iNKT cells (∼30%), whereas those from Jalpha18^−/−^ mice are devoid of iNKT cells. Transfer of liver iNKT cells from WT mice to Jalpha18^−/−^ mice resulted in clearly detectable tumor growth in the liver and spleen 10 days later, while tumor cells were undetectable in control mice receiving liver cells from Jalpha18^−/−^ mice (Figure 3). The differential effects of cells from WT versus Jalpha18^−/−^ mice strongly suggest that it is the presence or absence of iNKT cells that leads to the different outcomes of the two transfers.

We also reconstituted the iNKT cell compartment in Jalpha18^−/−^ mice by generating bone marrow chimeras. Jalpha18^−/−^ recipient mice were transplanted with either WT (C57BL/6) bone marrow to repopulate the iNKT cell population or with Jα18^−/−^ bone marrow as a control. After allowing for reconstitution of the immune system, mice were transplanted intravenously with 10^5^ TBL tumor cells. Mice receiving WT bone marrow had large numbers of tumor cells in the spleen, LN and livers 14 days later, while control mice receiving bone marrow from Jalpha18^−/−^ mice reduced tumor burdens in each of these organs (Figure S1). At the time of tumor analysis, partial restoration of iNKT cell numbers was observed in mice reconstituted with WT bone marrow but not in mice reconstituted with Jalpha18^−/−^ cells (data not shown). Together, these experiments are consistent with a role for iNKT cells in promoting lymphoma growth.

### CD8^+^ T Cells Mediate Effective Antitumor Immunity in the Absence of iNKT Cells

**Figure 4:**
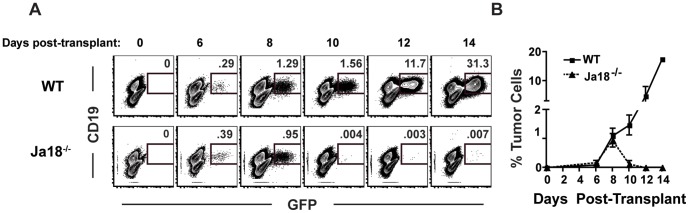
Tumor cells engraft in Jalpha18^−/−^ mice but are rejected. The frequency of tumor cells present among intrahepatic leukocytes was determined 6, 8, 10, 12, and 14 days after transplant in Jalpha18^−/−^ or WT mice. Data are (A) representative FACS plots and (B) combined data from 2 independent experiments (n = 2 for days 12 and 14, n = 3–7 for all other timepoints).

We next tested whether iNKT cells were directly promoting the growth of the lymphoma cells or if they were modulation the specific anti-tumor immune response. We compared tumor growth in Jαalpha18^−/−^ mice and WT mice beginning at day 6 post transplant and continuing every other day until day 14. We focused our analysis on the liver and spleen because, in WT mice, tumor cells are found in the liver and spleen at early time points and only accumulate in the LN after about day 10 post transplant (Figure S2). Tumor cells were readily detectable in the livers of both WT and Jalpha18^−/−^ mice 6 and 8 days after transplant, suggesting that the iNKT cells have little effect on the early stages of tumor growth (Figure 4). However, tumor cells were nearly undetectable in Jalpha18^−/−^ mice 10 days after transplant, while tumor growth progressed in WT mice (Figure 4A–B). This pattern of initial tumor growth followed by rapid regression is reminiscent of the immune-mediated tumor rejection observed in the MCA sarcoma model [19] and suggests that the reduced tumor growth in Jalpha18^−/−^, iNKT cell deficient, mice is due to immune-mediated tumor rejection that is otherwise ineffective when iNKT cells are present. Because Jalpha18^−/−^ mice were made on a 129sv/J genetic background prior to backcrossing to C57Bl6 [20], we analyzed tumor growth after transplant into B6x129 F1 mice to rule out lymphoma rejection caused by remaining minor histocompatibility differences that may exist between Jalpha18^−/−^ and C57BL6 strains. Tumor growth was equivalent in C57Bl6 and B6x129 F1 mice, suggesting that minor histocompatibility antigens are unlikely to be causing the rejection phenotype.

**Figure 5:**
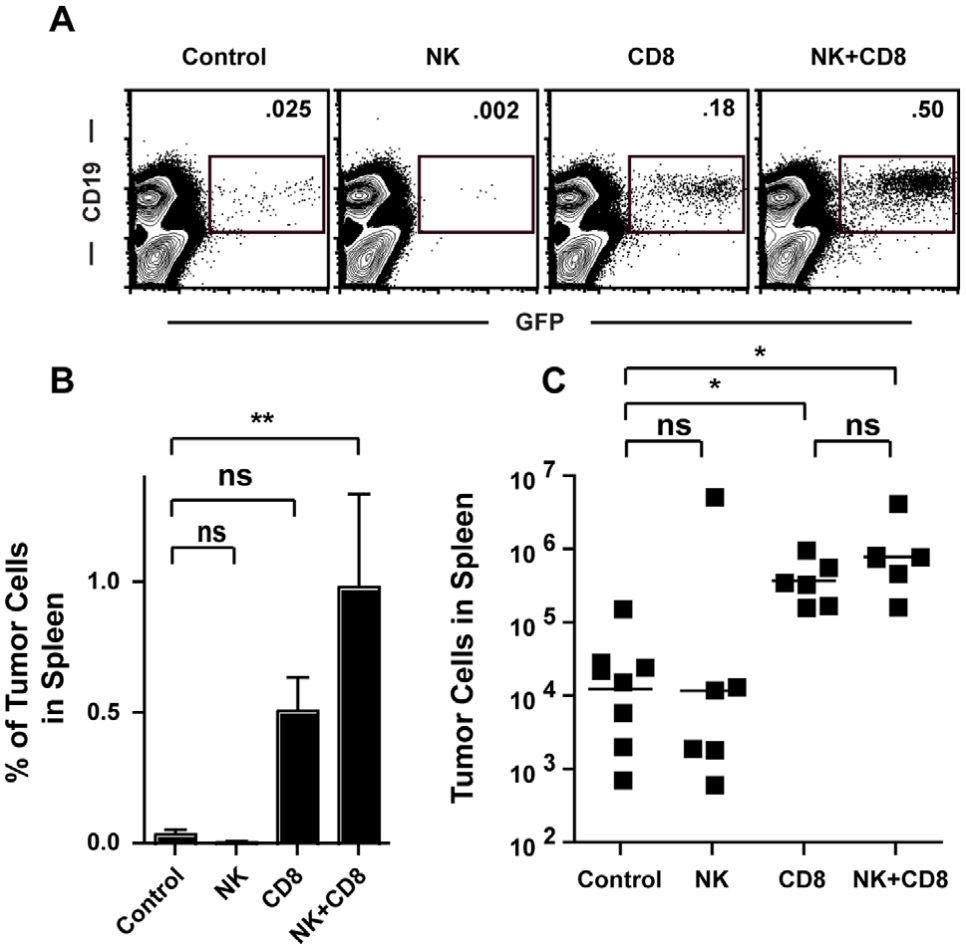
In the absence of iNKT cells, CD8^+^ T cells are required for tumor clearance. (A and B) The frequency of tumor cells in the spleens of tumor transplanted Jalpha18^−/−^ mice treated with control (anti-human DR1), anti-NK1.1, anti-CD8, or both anti-NK1.1 and anti-CD8 antibodies was measured by flow cytometry. Plots in (A) are gated on CD49b^-^NK1.1^-^CD4^-^CD8^-^ cells and the number on each plot indicates the frequency of tumor cells in that gate. (B) The frequency of tumor cells among splenocytes for the indicated antibody-treated Jalpha18^−/−^mice is shown. (C) The number of tumor cells present in spleens of antibody treated mice was determined (n = 6–8 pooled from 2 independent experiments). *p<.05. **p<.01.

CD8^+^ cytotoxic T cells and NK cells are important lymphocyte populations involved in the control of cancer by the immune system [21]. Loss of these cell types can result in outgrowth of dormant tumors or a failure to reject highly immunogenic tumors [19], [22]. To determine if these cell populations were responsible for tumor rejection in Jalpha18^−/−^ mice, we performed cell depletion experiments using monoclonal antibodies specific for NK cells or CD8^+^ T cells. Jalpha18^−/−^ mice were inoculated with TBL cells and were treated three and seven days later with depleting antibodies to either CD8^+^ T cells (anti-CD8), NK cells (anti-NK1.1), or both cell types simultaneously. Depletion was verified by flow cytometry at the time of analysis (data not shown). Depletion of NK cells alone had no effect on tumor growth compared with control antibody (anti-human DR1). CD8^+^ T cell depletion resulted in increased tumor growth compared to control treated or NK cell depleted mice (Figure 5A–B), indicating that CD8^+^ T cells are required for tumor rejection. Depletion of both CD8^+^ T cells and NK cells had no statistically greater effect on tumor cell accumulation than depletion of CD8^+^ T cells alone (Figure 5C), indicating that NK cells were not a significant contributor to tumor clearance in Jalpha18^−/−^ mice. Interestingly, while there was reduced tumor growth in CD3ε^−/−^ and TCRβ^−/−^ mice (Figure 1), which both lack iNKT cells and CD8^+^ T cells, this did not seem to be mediated by increased NK cell activity, as depletion of NK cells in TCRbeta^−/−^ mice had no effect on tumor growth (Data not shown). Therefore, it remains possible that some type of T cell may promote lymphoma growth via a mechanism distinct from iNKT cell suppression of the CD8^+^ T cell response.

### Lymphoma Specific CD8^+^ T Cell Responses are Enhanced in the Absence of iNKT Cells

**Figure 6:**
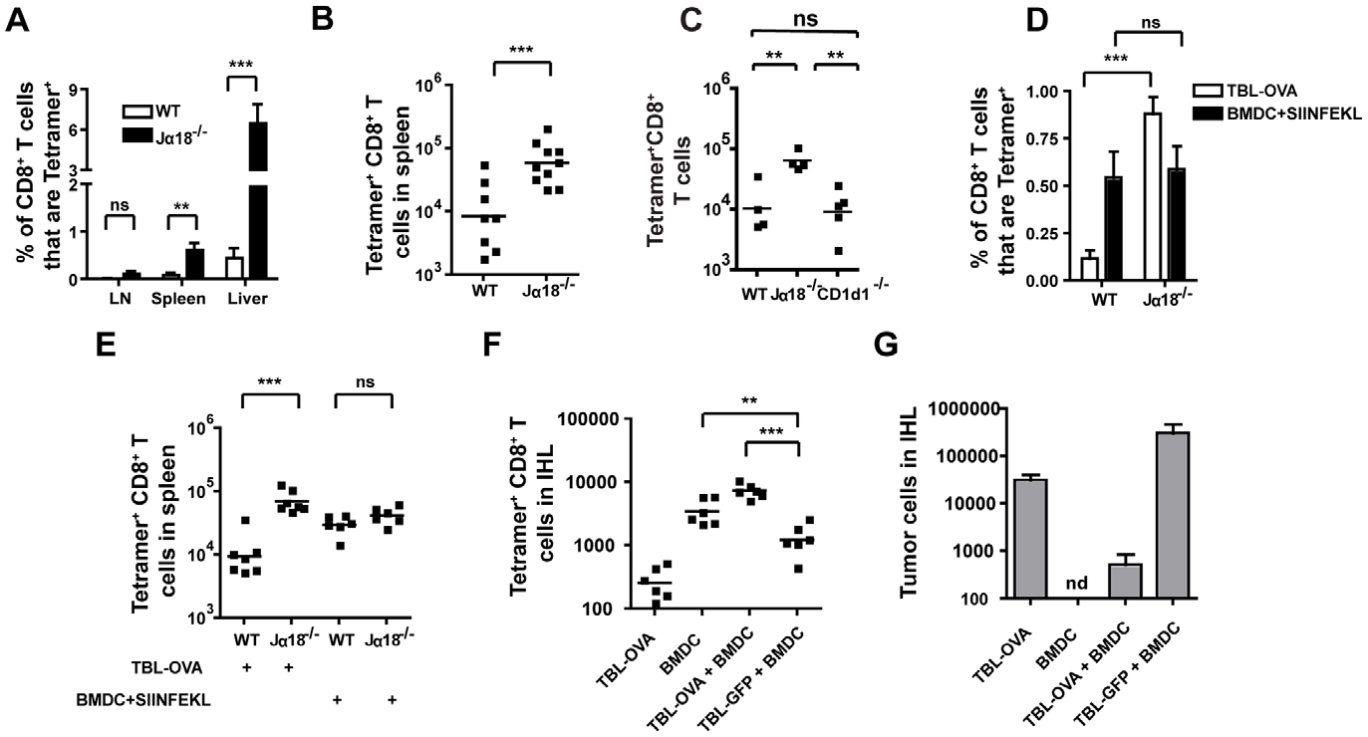
Jalpha18^−/−^ mice have enhanced CD8^+^ T cell responses to tumor-derived antigen. (A) The frequency of tumor reactive CD8^+^ T cells was determined by staining with K^b^/SIINFEKL tetramers. Staining was performed in the LN, spleen, and IHL 8 days after transplant of TBL-OVA tumor cells. (B) The number of K^b^/SIINFEKL reactive CD8^+^ T cells was determined by flow cytometry. (n = 8–10 pooled from two experiments). (C) WT, J**alpha**18^−/−^, and CD1d1^−/−^ mice were transplanted with 10^5^ TBL-OVA cells i.v. The number of K^b^/SIINFEKL-tetramer reactive CD8^+^ T cells in the spleen was determined by flow cytometry (One representative of three similar experiments is shown. n = 4–5/group. The J**alpha**18^−/−^ and WT groups in this experiment are also included in panel E, as the controls for these experiment were combined). (D and E) WT and J**alpha**18^−/−^ mice were given either 10^5^ TBL-OVA tumor cells or 10^5^ peptide pulsed BMDC intravenously. Spleen cells were analyzed 8 days later for the presence of K^b^/SIINFEKL tetramer reactive CD8^+^ T cells. The (D) frequency and (E) number of tetramer  =  CD8^+^ T cells is shown (n = 6–7 from two experiments). (F–G) The number of (F) K^b^/SIINFEKL reactive CD8^+^ T cells and (G) lymphoma cells was determined 8 days after administration of 10^5^ TBL-OVA cells, 10^5^ SIINFEKL-pulsed BMDC, or SIINFEKL-pulsed BMDC in combination with either TBL-OVA or TBL-GFP lymphoma cells. ***p<.001, **p<.01, nd  =  not detected.

To further explore the effects of iNKT cells on the anti-tumor CD8^+^ T cell response further, we engineered the TBL tumor cells to express the experimental antigen ovalbumin (TBL-OVA) by retroviral transduction. This allowed us to track CD8^+^ T cell responses to tumor cells using MHC class I tetramers [23]. OVA (tumor) specific CD8^+^ T cell responses on day 8 after tumor transplant were readily detectable in Jalpha18^−/−^ mice, whereas wild type mice had a significantly reduced proportion and number of OVA-specific CD8^+^ T cells (Figure 6A–B). Tumor specific T cells were present in the highest proportion among the intrahepatic leukocytes and were less frequent in the spleen (Figure 6A). Very few tumor-specific CD8^+^ T cells could be found in the LNs of either strain (Figure 6A), perhaps because few tumor cells have reached the lymph nodes by this early time point (Figure S1). These data are consistent with our earlier findings that CD8^+^ T cells reduce tumor burden in Jalpha18^−/−^ mice (Figure 5).

A comparison of the tumor growth phenotypes in CD1d^−/−^ and Jalpha18^−/−^ mice suggested that type II NKT cells may inhibit lymphoma growth in the absence of iNKT cells (Figure 2F). In support of this hypothesis, analysis of the tumor-specific CD8^+^ T cell response in CD1d1^−/−^ mice using TBL-OVA cells and K^b^/SIINFEKL tetramers indicated that the anti-tumor CD8^+^ T cell response was similar to that seen in WT mice (Figure 6C). This was in contrast to the response in Jalpha18^−/−^ mice, which was enhanced compared to WT controls (Figure 6C). In the absence of type II NKT cells, anti-tumor CD8^+^ T cells in CD1d1^−/−^ mice fail to fully expand in response to the tumor, and this response is numerically comparable to the suppressed CTL response seen in WT mice.

### Immunization at the Time of Tumor Transplant Overcomes iNKT-cell Mediated CTL Suppression

To find out whether the absence of iNKT cells affected the CD8^+^ T cell response to OVA in a context outside of lymphoma growth, we intravenously immunized WT and Jalpha18^−/−^ mice with either 10^5^ TBL-OVA cells or 10^5^ OVA-peptide pulsed bone marrow derived dendritic cells (BMDC) and assessed the OVA-specific CD8^+^ T cell response in the spleen 8 days later. The frequency of OVA-specific CD8^+^ T cells was identical in BMDC-immunized WT and Jalpha18^−/−^ mice, and was similar to the response seen in Jalpha18^−/−^ mice given TBL-OVA cells (Figure 6D–E). However, the frequency of OVA-reactive CD8^+^ T cells in TBL-OVA transplanted WT mice was significantly reduced compared with all other groups, suggesting that the lymphoma microenvironment is necessary for the suppressed CD8^+^ T cell response seen in WT mice. These results indicate that the decreased number of OVA-specific CD8^+^ T cells in tumor-bearing WT mice is due to tumor-induced modulation of the immune system (via iNKT cells) that can be reversed when iNKT cells are removed from the system.

In further support of this hypothesis, WT mice transplanted with TBL-GFP tumors and simultaneously immunized with SIINFEKL-pulsed BMDC showed a reduction in the number of anti-OVA CD8^+^ T cells compared to mice receiving only SIINFEKL-pulsed BMDC (Figure 6F). These results argue that the growth of the TBL tumor is able to suppress the immune response to BMDC immunization against an independent antigen. In contrast, when BMDC immunization with SIINFEKL peptide was simultaneous with TBL-OVA transplant, an enhanced CD8^+^ T cell responses and a reduction in lymphoma growth was induced (Figure 6F–G), indicating that immunization at the time of tumor transplant can induce a CD8^+^ T cell response that is sufficient to affect tumor burden. These data suggest that the lymphoma-induced immunosuppression by iNKT cells on tumor growth is a late event that is not present immediately after lymphoma transplant, therefore allowing for effective CTL priming and expansion by simultaneous BMDC immunization.

### Alpha-GalCer Administration Further Suppresses the CD8^+^ T Cell Response

**Figure 7:**
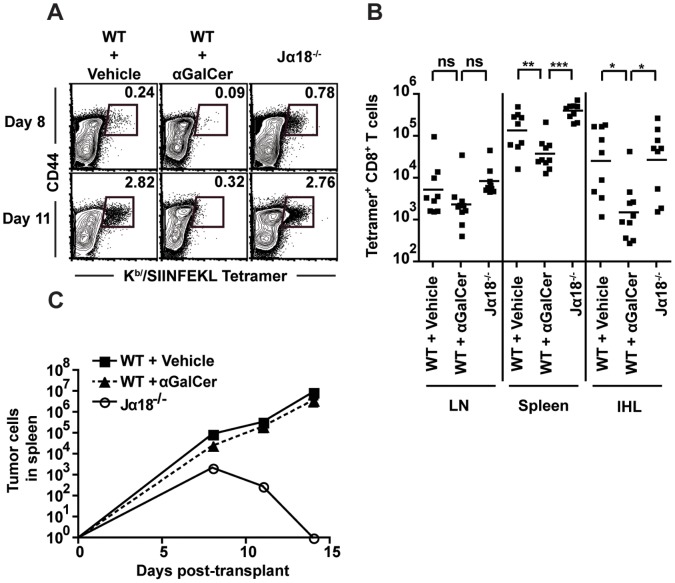
alphaGalCer enhances the suppressive effect of iNKT cells. WT mice were treated with either 1 microgram alpha-GalCer or vehicle control 1, 5, and 9 days after transplant of TBL-OVA tumor cells. Jalpha18^−/−^ mice were left untreated following tumor transplant. (A) The frequency of tumor-reactive CD8^+^ T cells was determined by staining with K^b^/SIINFEKL tetramers on days 8 and 11 after transplant. (B) The number of SIINFEKL-reactive CD8^+^ T cells was determined on day 11 in the LN, spleen, and IHL. (C) The number of TBL-OVA lymphoma cells in the spleen was determined by flow cytometry 8, 11, and 14 days after transplant in Jalpha18^−/−^, vehicle-treated WT, and alpha-GalCer-treated WT mice. Data are representative (A) FACS plots, (B) combined data from 3 independent experiments (n = 9–10), and (C) combined data from at least three independent experiments (n = 4–10/timepoint for both WT groups, n = 3–6 for Jα18^−/−^). *p<.05, **p<.01, ***p<.001.

Although the number of tumor specific CD8^+^ T cells in WT tumor-bearing mice was reduced 8 days after transfer of the tumor, this number was nearly the same in WT and Jalpha18^−/−^ mice 11 days after transplant (Fig. 7). These observations suggest that iNKT cells might delay the CD8^+^ T cell response to lymphoma antigens rather than inhibit it entirely. If true, stimulation of iNKT cells during tumor growth could potentially downregulate further the CD8^+^ T cell response, since iNKT cells suppress the CD8^+^ T cell response to tumor in WT mice.

To test this hypothesis, WT mice were given TBL-OVA tumor cells and treated with 1 µg alpha-GalCer 1,5, and 9 days after tumor transplant, and CD8^+^ T cell responses were analyzed on day 11, (Figure 7A). alpha-GalCer treatment reduced the number of tumor-specific T cells compared to vehicle treated controls (Figure 7A–B). The suppression of CD8^+^ T cell responses by alpha-GalCer was apparent in both the number (Figure 7B) and frequency (Figure 7A) of tumor-reactive CD8^+^ T cells. This effect was significant in both the spleen (p<.0001) and liver (p = .0002), while the differences in LNs followed a similar trend but did not reach statistical significance (p = .062). Finally, no significant difference in tumor growth between vehicle and alpha-GalCer-treated WT mice was observed (Figure 7C), suggesting that the CD8^+^ T cell response in vehicle-treated WT mice is insufficient to affect tumor growth. This is in line with our observations using CD8^−/−^ mice. Taken together, these data demonstrate that iNKT cells have the ability to suppress the anti-tumor CD8^+^ T cell response to Burkitt’s lymphoma.

## Discussion

In this study, we investigated the T cell response to a systemically disseminated B cell lymphoma model and found that iNKT and type II NKT cells are critical regulators of the antitumor CD8^+^ T cell response. We show that in the absence of iNKT cells, lymphoma cells expand in numbers at early time points but appear to be rejected 8–10 days after transplant. This rejection involves CD8^+^ T cells and allows for long-term tumor free survival in Jalpha18^−/−^ mice. CD8^+^ T cell responses to OVA-expressing lymphoma cells were vigorous in mice lacking only iNKT cells, were largely reduced in mice containing iNKT cells, and could be further limited in WT mice by the iNKT cell activating ligand alpha-GalCer. The suppressive effects of iNKT cells were observed only in the context of tumor growth and did not reduce CD8^+^ T cells responses to antigens in the absence of the tumor. Furthermore, our experiments with CD1d1^−/−^ mice suggest that type II NKT cells positively affect the anti-tumor CD8^+^ T cell response. In the absence of both iNKT and type II NKT cells, the tumor growth and the anti-tumor CD8^+^ T cell response are comparable to WT mice. Thus, in this system, immunity is mediated at least in part by tumor-specific CD8^+^ T cells, and the activity of these cells is regulated by iNKT and type II NKT cells.

Similar iNKT versus type II NKT cell antagonism has been noted before [13], [14], [24], [25], but our data differ from the published literature in several important ways. First, although iNKT – type II NKT cell counter-regulation has been posited in tumor systems, ours is the first report where iNKT cells are suppressive to anti-tumor immunity. Most significantly, when iNKT cell-mediated suppression is relieved, the anti-tumor response is sufficient to fully reject a tumor that is universally lethal in WT mice. It is unclear why this lymphoma initiates such a strong suppressive response by iNKT cells, although we hypothesize that several aspects that are unique to lymphoma may play a role.

iNKT cell mediated immunosuppression in lymphoma may reflect the localization of tumor growth within the secondary lymphoid organs, or may simply reflect the fact that the lymphoma developed through transformation of a lymphoid cell and is thus able to manipulate the immune response using mechanisms that are unavailable to other non-immune cell types. The identification of iNKT cell suppression of the CD8^+^ T cell response to lymphoma is in agreement with previous work demonstrating that iNKT cells are suppressive in several autoimmune models such as rheumatoid arthritis, experimental autoimmune encyphalomyelitis (EAE) [26], and type I diabetes [27] (reviewed in [2]). iNKT cell suppression of antitumor CD8^+^ T cell responses is consistent with these observations, as many tumor-associated antigens are self antigens. Because the B cell lymphoma used in the current study arose from an autoreactive B cell [16], it may still elicit the same suppressive pathways used to limit autoreactivity among normal B cells. Thus, the TBL lymphoma may simply be eliciting the immunosuppressive effects of iNKT cells that would normally occur during an autoreactive B cell response. Data from several autoimmune diseases suggests that non-Hodgkin’s lymphomas such as BL often arise from autoimmune settings [28], [29], and correlative evidence suggests that in human non-Hodgkin’s lymphoma, the preneoplastic state may present as an autoimmune polyclonal lymphoproliferative disease [30]. Lymphoma arising from these autoinflammatory conditions may be programmed such that the tumor activates the suppressive effects of iNKT cells much more strongly than other tumor types while minimizing the anti-tumor functions of iNKT cells elicited by many solid tumors. Immunization at the time of lymphoma transplant was able to overcome iNKT cell mediated suppression, suggesting that manipulation of the cytokine environment during early tumor growth can alter or overcome the effects of iNKT cells.

The data here present an apparent paradox: that the tumor grows at the same rate whether or not CD8^+^ T cells are present, thus CD8^+^ T cells do not affect the growth of the tumor in normal mice. However, in the absence of iNKT cells tumor growth is severely inhibited by the action of CD8^+^ T cells. How can CD8^+^ T cells have a detectable role in one situation and not the other? We interpret these data as indicative of the effectiveness of iNKT cell mediated immunosuppression. In normal animals, the iNKT cells somehow completely inhibit the response of CD8^+^ T cells, therefore the absence of the latter cells in normal animals is not detectable. When inhibition by iNKT cells is absent, the CD8^+^ T cells are freed to expand and act on the tumor cells. Previous studies have shown that tumors originating from WT mice can grow equally well when transplanted into either WT or Rag2^−/−^ mice [19], suggesting that the processes of immunoediting can produce a tumor that is independent of CD8^+^ T cell control. We hypothesize that in the TBL system used here, rather than being selected for lower antigenicity through immunoediting, the TBL lymphoma escapes CD8^+^ T cell control through the induction of iNKT cell mediated immunosuppression.

Studies in a variety of both spontaneous and transplantable solid tumor models [31]–[33] have combined to establish a role for iNKT cells in activating anti-tumor immunity, primarily through activation of NK and CD8^+^ T cells [34]. Because, to date, no appropriate markers can be used for the unequivocal identification of type II NKT cells, these cells have been less well studied than iNKT cells, and knowledge of their function is largely inferred from comparisons of phenotypes in Jalpha18^−/−^ and CD1d1^−/−^ mice. The majority of data from solid tumor models suggests that type II NKT cells suppress the anti-tumor response [2], [12]–[14]. The mechanism for this suppressive effect is generally thought to be mediated by inducing a Th2 cytokine bias through secretion of cytokines such as IL-4 and IL-13. However it is also apparent that other unknown mechanisms, distinct from altering in the Th1/Th2 cytokine balance, might also be involved [2], [35]. In contrast to their suppressive functions in solid tumors, our data suggest that type II NKT cells are activating in the anti-lymphoma immune response. Development of better techniques for type II NKT cell identification and function would greatly aid in understanding their function the cancer microenvironment.

While the role of iNKT cells as activators of antitumor immunity has been well documented for solid tumors, evidence that they function similarly in B cell lymphomas is lacking, although there are published examples [14]. In contrast, our data suggest an opposite role for iNKT cells in modulating the immune response to B cell lymphoma; that iNKT cells suppress anti-tumor immunity. The suppression of anti-lymphoma immunity by iNKT cells was previously observed using the RMA/S T cell lymphoma, but was in that case dependent on experimentally increasing expression of CD1d on lymphoma cells, which lead to a modest increase in overall survival of CD1d^−/−^ and Jalpha18^−/−^ mice compared to WT mice following tumor challenge [36]. Our data illustrate a much more potent effect of iNKT cell loss, where the majority of iNKT cells remain tumor free while all WT mice develop lethal tumors. Suppressive effects of iNKT cells in autoimmunity are generally thought to be caused by production of the Th2 cytokines IL4 and IL-13, while their immunostimulatory effects in other tumor systems are typically IFNγ-dependent (reviewed in [2]). It follows that the simplest hypothesis for the iNKT cell dependent CD8^+^ T cell suppression in the TBL model is the secretion of large amounts of IL-4 and IL-13, which biases the immune system away from a cytotoxic Th1 response. However, administration of alpha-GalCer to tumor bearing mice enhances the ability of iNKT cells to suppress, despite the fact that alpha-GalCer induces strong expression of both IFN-gamma and IL-4 from iNKT cells.

Ours and previously published data [36], [37] [38] suggests that, like conventional CD4^+^ T cells and type II NKT cells, iNKT cells have the capacity to suppress anti-tumor immunity. This iNKT cell-mediated suppression may have been predicted based on the established ability of type II NKT cells to perform this same function [12], [13], [35], [39]. Because iNKT cells and type II NKT cells share many common phenotypic and functional features [2], [5], it is not entirely surprising that, under varying conditions present in different tumor microenvironements, both NKT subtypes can perform similar suppressive functions. Our data suggests a role reversal for type II NKT cells. While type II NKT cells generally suppress anti-tumor immune responses [13], [14], the data presented here suggests that they are also capable of supporting the anti-tumor response. In the TBL lymphoma model, type II NKT cells appear to be necessary for effective anti-tumor immunity, but the effects of these cells is limited by the dominant, suppressive effects of iNKT cells. Alternatively, changes to the TCR repertoire of conventional alpha-beta-T cells in Jalpha18^−/−^ mice could account for the tumor rejection seen in this strain. Disruption of normal TCRalpha gene segment utilization in Jalpha18^−/−^ mice (that is independent of iNKT cells) may alter composition of the T cell compartment such that the balance of the antitumor response is shifted away from immunosuppression and tumor growth and toward CD8^+^ T cell mediated rejection. Thus alterations in the conventional T cell repertoire could alternatively explain the tumor phenotype differences observed between Jalpha18^−/−^ and CD1d1^−/−^ mice, as the latter strain also lacks iNKT cells but has a conventional alpha-beta-T cell repertoire that is comparable to WT mice.

The direct function of T cells in controlling human BL is unclear. The occurrence of immunodeficiency associated BL suggests that the adaptive immune system is important in preventing or suppressing lymphomagenesis, but there is little direct evidence of T cell control of human BL. Although T cells are thought to be important for graft-versus-leukemia and graft-versus-lymphoma (GVL) effects following allogeneic stem cell transplantation, evidence for a significant GVL effect for Burkitt’s lymphoma is incomplete [40]–[41]. This is likely due in part to the efficacy of current aggressive treatment regimens (reviewed in [42]), which greatly reduce the need for additional treatment strategies.

Understanding the cellular elements that regulate anti-tumor responses has the potential to reveal new targets for tumor immunotherapy. Our analysis of the T cell response to a transplanted B cell lymphoma identified NKT cells as critical regulators of the anti-tumor CD8^+^ T cell response. Determining why some cancers induce immunosuppressive NKT cell phenotypes (mediated by either iNKT or type II NKT cells) while other cancers may induce predominantly anti-tumor NKT cell effects is a major unanswered question in the tumor immunology field. Understanding the function of NKT cells, particularly iNKT cells, in the lymphoma environment is important in designing tumor immunotherapy whether or not the therapy is specifically targeting iNKT cells. Experimental analysis of iNKT cell function in lymphoma rather than solid tumor growth is lacking, and the data presented here suggest a pro-rather than anti-tumor function for this cell type. Our results identify iNKT cell depletion or inhibition as a potential immunotherapy target for Burkitt’s lymphoma.

## Materials and Methods

### Mice

C57BL6 mice were purchased from The Jackson Laboratory (Bar Harbor, ME) or were bread locally at the National Jewish Health (NJH). CD3epsilon^−/−^, Jalpha18^−/−^, CD1d1^−/−^, TCRbeta^−/−^ CD4^−/−^, CD8^−/−^, and TCRdelta^−/−^ were bred and housed at NJH. Jalpha18^−/−^ were provided by Masaru Taniguchi and CD1d1^−/−^ were provided by Luc van Kaer (Vanderbuilt University). Bone marrow chimera experiments were performed by ablating the immune system of recipient mice with 900 rads of gamma-irradiation. Following irradiation, mice were reconstituted with freshly isolated bone marrow from the tibias and femurs of either WT or Jalpha18^−/−^ donor mice. All tumor experiments were performed at least 8 weeks after bone marrow transplant to allow for reconstitution of the immune system. For reconstitution of Jalpha18^−/−^ mice by transfer of peripheral iNKT cells, we pooled intrahepatic leukocytes (IHL) from C57Bl/6J mice and transferred them to Jalpha18^−/−^ recipients via tail vein injection. Due to technical limitations, we were unable to isolate sufficient numbers of purified iNKT cells for reconstitution. IHL pooled from WT mice contained between 6×10^5^ and 1.5×10^6^ iNKT cells, depending on the experiment. Control Jalpha18^−/−^ recipients received an equal number of total IHL isolated from Jalpha18^−/−^ donors, which contained no iNKT cells. All mice were maintained in specific pathogen free conditions in the National Jewish Health AALAC accredited facility. All experiments involving animals were approved by the National Jewish Health Institutional Animal Care and Use Committee (IACUC) (protocol #AS2640).

### Tumor Cell Line

The generation of B-cell lymphoma cell lines used in these experiments has been previously described [16]. The tumor cell line TBL-12 from the E-mu-MYC/BCR^HEL^/sHEL mice was used for tumor transplant experiments. To facilitate differentiation of malignant cells from endogenous B cells, we transduced the TBL-12 cells with a retrovirus encoding GFP. GFP-expressing tumor cells displayed in vitro and in vivo growth rates that were comparable to nontransduced cells (unpublished data). In vivo experiments were performed by transplanting 10^5^ GFP-expressing TBL-12 cells. Tumors formed with 100% penetrance in WT mice. After transplant tumor growth that was initially located primarily in the spleen and liver (Figure S1). GFP^+^ tumor cells could be found in the lymph nodes around 10 days after transplant, and rapidly expanded from days 10–14 (Figure S1).

### Dendritic Cell Immunizations and AlphaGalCer Treatment

Bone marrow dendritic cells (BMDC) were produced by flushing bone marrow from femurs and tibia with balanced salt solution (BSS). Red blood cells were lysed with buffered ammonium chloride and cells were cultured with GM-CSF for seven days, with media + GM-CSF being refreshed on days 2–3 and 5–6. BMDC were activated on day 7 with 50 ng/ml LPS for 2 hours prior to addition of OVA peptide (SIINFEKL) for an additional 2 hours. Cells were washed extensively prior to transfer to recipient mice via tail vein injection. 10^5^ peptide-pulsed BMDC were transferred per recipient. For experiments in which mice were immunized with BMDC and transplanted with lymphoma cells, tumor cells were transplanted either immediately or 6 hours after the immunization, depending on the experiment. For in vivo activation of iNKT cells, 1 microgram alphaGalCer (KRN7000, from Funakoshi) in 250 microliter was administered i.p. to WT mice 1, 5, and 9 days after tumor transplant. Control mice were administered an equivalent volume of vehicle at the same time points.

### Tumor Free Survival Experiments

To assess tumor free survival, WT and Jalpha18^−/−^ mice were transplanted with 10^5^ TBL-GFP cells. Mice were observed daily for indication of tumor formation as indicated by hunched posture, labored breathing, lethargy, and palpable LN tumors. Mice were euthanized when tumor symptoms became apparent. Mice were monitored for at least 50 days.

### Cell Preparation

Lymph nodes and spleens were prepared for flow cytometry by disruption through nylon mesh prior to red blood cell lysis by buffered ammonium chloride. The number of live cells was determined by trypan blue exclusion or by counting cell nuclei using a Coulter Counter. Intrahepatic leukocytes (IHL) were prepared by mashing livers through 40 micrometer filters and washing twice in BSS. IHL were then isolated by centrifugation on a discontinuous (40%/60%) percoll (Sigma) gradient for 20 minutes at 900×G at room temperature.

### Antibody Depletion

Monoclonal antibodies (mAb) to NK1.1 (PK136), CD8α (53−6.72), or control antibody to human DR1 (20LC-11.1) were purified from the appropriate hybridoma supernatants using protein G. Jalpha18^−/−^ mice were injected with 250 microgram anti-CD8, 250 µmicrogram control mAb, or 500 micrograms anti-NK1.1 four and seven days after tumor transplant. Mice were sacrificed on day 10 after transplant and the number of tumor cells was determined in the spleen and liver by flow cytometry. Depletion of CD8 T cells and NK cells was verified by staining with CD8beta and CD49b antibodies, respectively.

### Flow Cytometry

Flow cytometry was performed on Cyan (BD) or Facscan (BD) instruments, and data was analyzed using FloJo software (Treestar, Inc.). MHC class I tetramer staining was performed by incubating cells with fluorescently labeled tetramer at 37°C for two hours prior to the addition of surface labeling antibodies conjugated to PE, PerCP-Cy5.5, PE-Cy7, Pacific Blue, APC, Alexa-Fluor 647, eFluor 450, APC-Cy7, and APC-eFluor780. The following antibody conjugates from eBioscience were used: CD4 (RM 4–5), CD8 (53−6.7), CD19 (1D3), CD11b (M1/70), CD3 (145-2C11), NK1.1 (PK136), F4/80 (BM8), Gr1 (RB6-8C5), CD44 (1M7), B220 (RA3-6B2). MHCII (Y3P) was produced and conjugated in house. Samples were incubated on ice for 30 minutes and were washed twice prior to analysis. All staining was performed in BSS with normal mouse serum and anti-Fc gamma RIIb (24G2) antibody to block non-specific antibody binding.

### Retroviral Transduction

Chicken ovalbumin (OVA) cDNA was subcloned in to an MSCV-IRES-Thy1.1 retroviral expression vector and transfected into Phoenix cells using Effectene transfection reagent (Qiagen). Supernatants containing retroviruses were used to transduce tumor cells as previously described, and Thy1.1+ transduced cells were magnetically sorted using PE-labelled anti-Thy1.1 antibody (eBioscience) and anti-PE microbeads (Miltenyi Biotech). Expression of Ovalbumin was confirmed by flow cytometric staining using the 25D1.16 antibody, which recognizes the SIINFEKL epitope of OVA presented by the MHCI K^b^ molecule.

### Statistical Analysis

All statistical analysis was performed using Prism Graphpad software (Graphpad software, Inc.). For experiments with 2 groups the student’s T test was used. For experiments with 3 or more groups data was analyzed with a one-way analysis of variance and Tukey post-test. For data presented on a log scale, all statistical analysis was performed on log-transformed data.

## Supporting Information

Figure S1 **Tumor growth for TBL lymphoma cells.** WT mice were transplanted with 10^5^ lymphoma cells i.v. Tumor growth was assessed in the LN, spleen, and liver (intrahepatic leukocytes, IHL) on days 6, 8, 10, 12, and 14 post-transplant. Data are (A) representative FACS plots or (B) quantitation of 3 independent experiments.
(TIF)

Figure S2 **Reconstitution of iNKT cells in Jalpha18^−/−^ mice restores tumor growth.** Lethally irradiated Jalpha18^−/−^ mice were reconstituted with either WT or control Jalpha18^−/−^ bone marrow. After ∼8 weeks, we inoculated mice with 10^5^ TBL-GFP cells intravenously. (A) The number of tumor cells among LN, spleen, and IHL was determined 14 days after transplant by flow cytometry. (n = 2–4 from one representative of 3 experiments).
(TIF)
