# Supplementary figures and images for: iNKT Cells Suppress the CD8+ T Cell Response to a Murine Burkitt’s-Like B Cell Lymphoma
Source: PLoS One. 2012 Aug 7;7(8):e42635. doi: 10.1371/journal.pone.0042635 (PMC3413636; doi:10.1371/journal.pone.0042635)

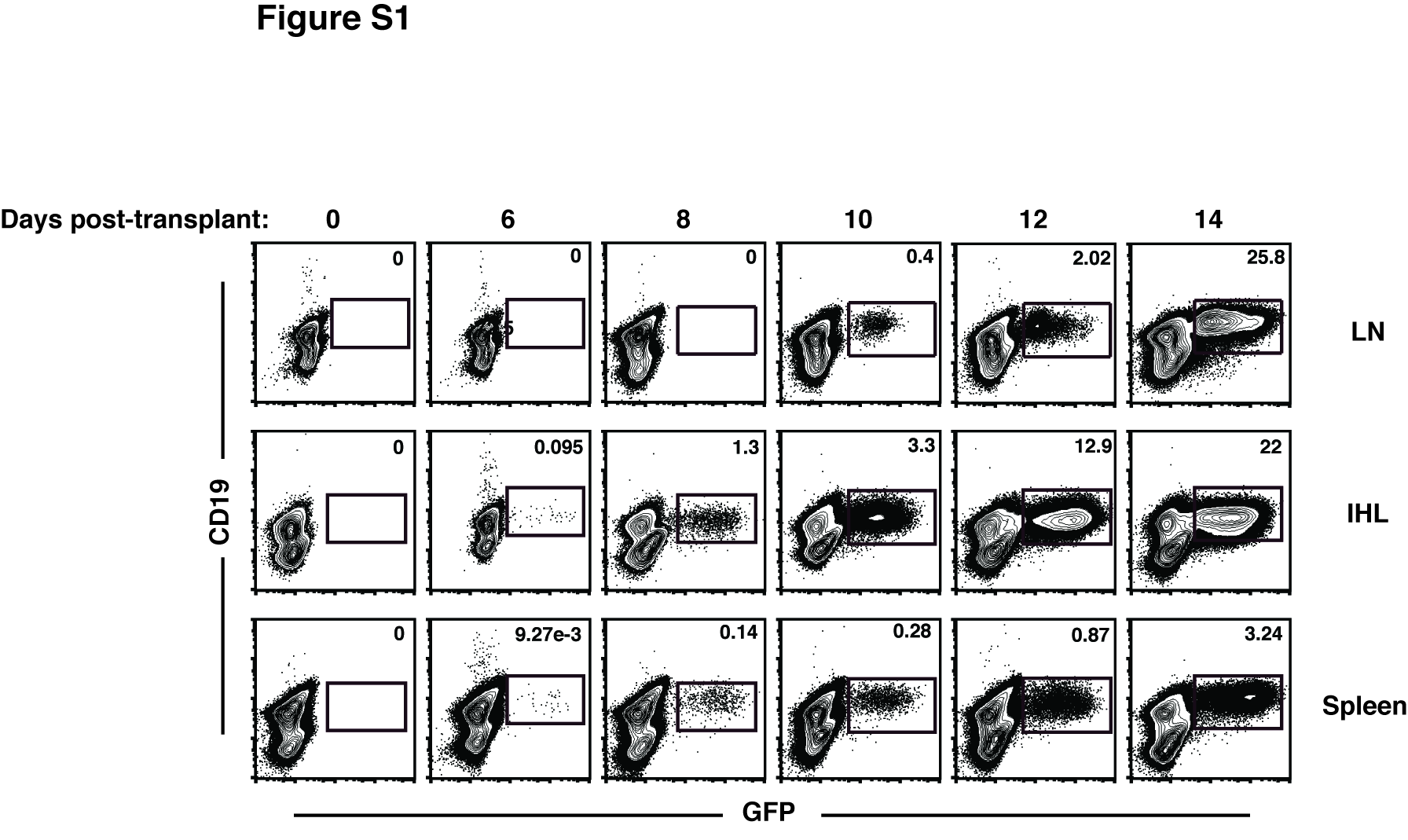

Supplement: Figure S1 — Tumor growth for TBL lymphoma cells. WT mice were transplanted with 105 lymphoma cells i.v. Tumor growth was assessed in the LN, spleen, and liver (intrahepatic leukocytes, IHL) on days 6, 8, 10, 12, and 14 post-transplant. Data are (A) representative FACS plots or (B) quantitation of 3 independent experiments. (TIF) [file pone.0042635.s001.tif]

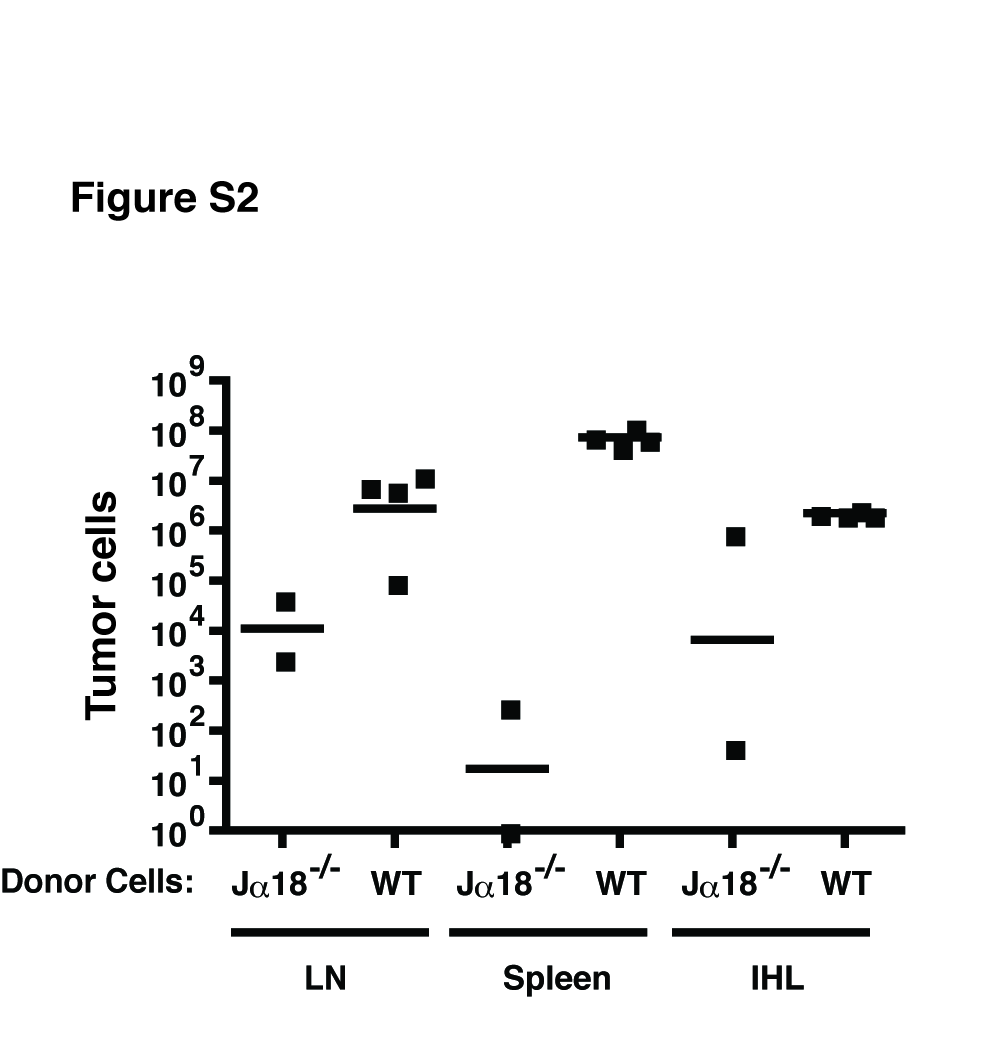

Supplement: Figure S2 — Reconstitution of iNKT cells in Jalpha18−/− mice restores tumor growth. Lethally irradiated Jalpha18−/− mice were reconstituted with either WT or control Jalpha18−/− bone marrow. After ∼8 weeks, we inoculated mice with 105 TBL-GFP cells intravenously. (A) The number of tumor cells among LN, spleen, and IHL was determined 14 days after transplant by flow cytometry. (n = 2–4 from one representative of 3 experiments). (TIF) [file pone.0042635.s002.tif]
